# Metabolomic characterization of sunflower leaf allows discriminating genotype groups or stress levels with a minimal set of metabolic markers

**DOI:** 10.1007/s11306-019-1515-4

**Published:** 2019-03-30

**Authors:** Olivier Fernandez, Maria Urrutia, Thierry Berton, Stéphane Bernillon, Catherine Deborde, Daniel Jacob, Mickaël Maucourt, Pierre Maury, Harold Duruflé, Yves Gibon, Nicolas B. Langlade, Annick Moing

**Affiliations:** 1 UMR1332 Biologie du Fruit et Pathologie, INRA, Centre INRA de Bordeaux, 71 av Edouard Bourlaux, 33140 Villenave d’Ornon, France; 2 UMR AgroImpact, INRA, Estrées-Mons, 80203 Péronne, France; 3 Plateforme Métabolome Bordeaux, CGFB, MetaboHUB-PHENOME, 33140 Villenave d’Ornon, France; 4 UMR LIPM, INRA, CNRS, Université de Toulouse, 31326 Castanet-Tolosan, France; 5 Present Address: Laboratoire RIBP, Université de Reims Champagne Ardenne, Moulin de la Housse Chemin des Rouliers, 51100 Reims, France; 6 Present Address: Enza Zaden Centro de Investigacion S.L., Santa Maria del Aguila, 04710 Almeria, Spain; 7 Present Address: Centre for CardioVascular and Nutrition, UMR INRA-INSERM, Aix-Marseille Univ, INSERM, 13005 Marseilles, France

**Keywords:** Metabolic markers, Metabolomics, Sunflower, Water stress, Maintainer–restorer lines

## Abstract

**Introduction:**

Plant and crop metabolomic analyses may be used to study metabolism across genetic and environmental diversity. Complementary analytical strategies are useful for investigating metabolic changes and searching for biomarkers of response or performance.

**Methods and objectives:**

The experimental material consisted in eight sunflower lines with two line status, four restorers (R, used as males) and four maintainers (B, corresponding to females) routinely used for sunflower hybrid varietal production, respectively to complement or maintain the cytoplasmic male sterility PET1. These lines were either irrigated at full soil capacity (WW) or submitted to drought stress (DS). Our aim was to combine targeted and non-targeted metabolomics to characterize sunflower leaf composition in order to investigate the effect of line status genotypes and environmental conditions and to find the best and smallest set of biomarkers for line status and stress response using a custom-made process of variables selection.

**Results:**

Five hundred and eighty-eight metabolic variables were measured by using complementary analytical methods such as ^1^H-NMR, MS-based profiles and targeted analyses of major metabolites. Based on statistical analyses, a limited number of markers were able to separate WW and DS samples in a more discriminant manner than previously published physiological data. Another metabolic marker set was able to discriminate line status.

**Conclusion:**

This study underlines the potential of metabolic markers for discriminating genotype groups and environmental conditions. Their potential use for prediction is discussed.

**Electronic supplementary material:**

The online version of this article (10.1007/s11306-019-1515-4) contains supplementary material, which is available to authorized users.

## Introduction

Sunflower (*Helianthus annuus* L.) is the fourth major crop providing seed for oil production worldwide. In 2016, world production reached 45 MT from 26 Mha, principally in Europe (around 70%), Ukraine being the world leader (Oilworld 2016; Hussain et al. 2018). Worldwide production has increased constantly ever since (Oilworld 2016). Sunflower accounts for more than 50% of total world table-oil consumption. Additionally, its high biodegradability makes it suitable for non-alimentary uses such as in paints and bioplastics.

Native to North America and introduced into Europe in the sixteenth century, sunflower became a major crop in this area in the early 1960s. Further development was achieved after the introduction of hybrid varieties in the early 1980s. Hybrid varieties are based on the use of cytoplasmic male-sterile (CMS) lines (Vear 2016), like many other crops (Chen and Liu 2014). The male sterility used for sunflower hybrid production, called PET1-CMS, was first identified from an interspecific cross between *Helianthus petiolaris* and *H. annuus*. It results from the reorganization of mitochondrial DNA that generated a new open reading frame ORFH522 co-transcribed with apt1 gene and coding a 16 kDa protein. This leads to modified mitochondrial functions and affects pollen development (Balk and Leaver 2001) through a decline in the mitochondrial membrane integrity and the respiratory control ratio. The mitochondrial protein ORFH522 appears to be expressed in all tissues, but the deleterious phenotype associated with PET1-CMS has been thought to be limited to the anthers, and no apparent extra phenotypes have been found in other organs (Horn and Friedt 1999; Balk and Leaver 2001).

To complement the mutational effect, a nuclear restoration gene (noted *Rf1*) is used in sunflower hybrid production. Restoration genes are nuclear and generally encode tetratricopeptides that are thought to transcriptionally control the *CMS* mitochondrial gene (Chen and Liu 2014; Igarashi et al. 2016; Yu et al. 2016). Finally, sunflower hybrid production is based on crossing a restorer line called R bearing a functional restoration allele *Rf1* (that recovers the PET1-CMS male-sterility phenotype) to a male-sterile PET1-CMS line called A (carrying a recessive *rf1* allele). To maintain this male-sterile line, a maintainer line called B, isogenic to the A line, is also used. Each B line carries the *rf1* allele but is male-fertile, as it does not carry the CMS-PET1 cytoplasm. Therefore, the B line is widely used for phenotypic and agronomic description of the line.

Since the introduction of hybrid varieties, sunflower has undergone an active breeding process (Vear 2016), mainly thanks to molecular marker-assisted selection. Hybrids have been selected with increased resistance to downy mildew (Qi et al. 2016), sclerotinia (Talukder et al. 2014) and water stress (Marchand et al. 2013; Owart et al. 2014), although sunflower is often cited as moderately drought-tolerant (Hussain et al. 2018). This selection process will benefit from the recent sequencing of the maintainer inbred line XRQ (Badouin et al. 2017). As part of these selection efforts, our group is currently involved in searching for metabolic markers of sunflower performance. A definition of biomarkers (and their sub-category metabolic markers) emerged from the field of medicine as a characteristic objectively measured to indicate a given biologic, pathologic or pharmacologic response (Fernandez et al. 2016). In plant science, metabolic markers have been defined as metabolites or groups of metabolites that are measured to predict or discriminate plant responses or performance (Fernandez et al. 2016). The use of metabolic markers to predict criteria of plant performance is recent, with pioneering papers dating from the early 2010s (Meyer et al. 2007; Riedelsheimer et al. 2012). The possibilities offered by these markers in plant selection processes were reviewed recently and a pipeline to search and use them has been proposed (Fernandez et al. 2016). The authors emphasized that the search for metabolic markers requires a first step of analysis on a small core set of genotypes. The present article investigates this first step, which includes (1) testing the analytic pipeline to establish the dynamic range of targeted metabolites, (2) confirming the presence of several secondary or “specialized” metabolites (as defined by Hartmann 2007; Pichersky and Lewinsohn 2011) and (3) investigating which metabolites are essential for differentiating groups of samples such as, in our case, water treatment (well-watered, WW, vs. drought-stressed, DS) and line status (maintainer, B, vs. restorer, R). These metabolites could later serve as metabolic markers. Furthermore, we tested different statistical methods for variable selection in order to find the best and smallest sets of metabolite markers. Indeed, for a given agronomical trait, the deployment of metabolic markers among breeders will depend on their cost (Fernandez et al. 2016).

For this purpose, we used a combination of targeted and untargeted metabolomic analyses on sunflower leaf samples obtained from B or R lines and in WW and DS conditions. Our results show that a limited number of markers can clearly differentiate WW from DS samples and in a more discriminant manner than the physiological data presented in Blanchet et al. (2018), which are classically used to discriminate individuals subjected to DS. To our surprise, another leaf metabolic marker set was able to discriminate B lines from R ones. Our data underline the potential of metabolic markers for discriminating genotypes and environmental conditions. Their potential use in sunflower breeding for performance prediction is discussed.

## Materials and methods

The protocols used are detailed in Online Resource 1 and summarized here.

### Plant material and growth conditions

The experiment was performed in 2013 in the phenotyping platform “Heliaphen” (Gosseau et al. 2018). Eight sunflower lines, four B and four R lines, were grown in two conditions (WW and DS) with three replicates, leading to a total of 48 samples. Irrigation was stopped at 38 days after germination (DAG; Schneiter and Miller 1981) for DS plants. Soil evaporation was estimated according to Marchand et al. (2013). Both WW and DS plants were weighed four times per day by the Heliaphen robot to estimate plant transpiration (Gosseau et al. 2018). At 47 DAG, leaves for metabolomic analyses were harvested without their petiole and frozen in liquid nitrogen. Two other leaves (mature and young leaves) were harvested for physiological trait measurements. During the experiment, two samples were excluded before leaf sampling (excessive irrigation was detected when analysing final Heliaphen readings) and four samples could not be analysed because of insufficient powder quantity. This resulted in a total of 42 samples submitted to metabolic analyses.

### Physiological trait measurements for plant phenotyping

Plant and leaf physiological data are part of a larger dataset presented in Blanchet et al. (2018). Specific leaf area (SLA) was determined according to Allinne et al. (2009). Both leaf osmotic potential (OSM_POT) and leaf osmotic potential at full turgor (OSM_POT_100) were measured as described in Poormohammad Kiani et al. (2007). To assess carbon isotope discrimination (CID), samples were oven-dried, ground, weighed and analysed using a continuous low isotope ratio mass spectrometry at the Stable Isotope Platform SHIVA (University of Toulouse, France).

### Targeted compound measurements

For each sample, about 20 mg fresh weight were extracted as in Hendriks et al. (2003). Sucrose, glucose, and fructose (Jelitto et al. 1992), malate (Nunes‐Nesi et al. 2007), citrate (Tompkins and Toffaletti 1982) and glucose-6-P (Gibon et al. 2002) were determined in the ethanolic supernatant. Starch (Hendriks et al. 2003) and protein (Bradford 1976) contents were determined on the pellet. Assays were carried out in 96-well microplates.

Individual free amino acid analysis was carried out using an UPLC separation with fluorescent detection after derivatization using 6-aminoquinolyl-*N*-succinimidyl carbamate (AQC)-tag (a method hereafter referred to as UPLC-Fluo).

For lipid analysis, fatty acid methyl esters (FAMES) were measured after hydrolysis of 20 mg dry weight (DW) with 2.5% H_2_SO_4_ (v/v) in methanol. GC-FID was performed using an Agilent 7890 gas chromatograph (Agilent, Santa Clara, California) equipped with a Carbowax column (15 m × 0.53 mm, 1.2 µm; Alltech Associates, Deerfield, IL, USA) and flame ionization detection. FAMES were identified by comparing their retention times with commercial fatty acid standards (Sigma, Saint-Quentin Fallavier, France) and quantified using ChemStation (Agilent).

### ^1^H-NMR analysis of major polar compounds

Polar metabolites were extracted from lyophilized powder (40 mg DW per biological replicate) with an ethanol–water series (80/20, 50/50, 0/100 v/v) at 80 °C as described in Deborde et al. (2009) with modifications. This three-step extraction process (ethanol–water series) was chosen to take into account the diverse affinities and solubilities of leaf major polar compounds (i.e. sugars, organic acids, amino-acids) for ethanol or water, in order to obtain an accurate view of these compounds in leaf extracts. The 1D (cpmg and single-pulse) spectra were processed using the NMRProcFlow application v1.1 (Jacob et al. 2017; http://nmrprocflow.org/). For the cpmg dataset, this resulted in 479 normalized variables corresponding to spectral regions (named Unk_ppm:number in Online Resource 2) which included compounds that were annotated later on. The assignments of metabolites in the ^1^H-nuclear magnetic resonance (NMR) spectra were made by comparing the proton chemical shifts with public or local spectral databases and by spiking the samples with the corresponding commercial compounds. 2D experiments were performed on a representative selected extract taken from the WW condition. Quantification of 11 identified compounds was performed by using quantified single-pulse spectra dataset and calibration curves.

### LC–ESI–QTOF–MS untargeted analysis of semi-polar metabolites

Liquid chromatography–electrospray-ionization–time-of-flight–mass spectrometry (LC–ESI–QTOF–MS) profiling of aqueous methanol extracts containing 0.1% formic acid was performed with extracts obtained from 20 mg DW lyophilized powder. An Ultimate 3000 HPLC (Dionex, Sunnyvale, CA, USA) was used to separate metabolites on a reversed-phase C18 column using an acetonitrile gradient in acidified water. Metabolites were detected by using a hybrid quadrupole/time-of-flight mass spectrometer (micrOTOF-*Q*, Bruker Daltonics, Bremen, Germany). Electrospray ionization in positive mode was used to ionize the compounds. A quality control sample (QC) was injected after each set of ten samples. The MS data were processed using XCMS (Smith et al. 2006) and R scripts for filtering. A total of 1519 features were detected and reduced to 540 metabolic variables after filtering. The corresponding MS-based variables were named using their nominal masses in dalton and retention time in seconds in Online Resource 2 (MxxxTyyy). Metabolite identification was performed using the accurate-mass data and Orbitrap (Thermo Fisher, Villebon-sur-Yvette, France) MS and MS/MS data of a representative sample extract.

### Statistical analyses

All statistical analyses were performed using the R Software (http://www.r-project.org/), the R package mixOmics (Rohart et al. 2017) and the BioStatFlow online tool (biostatflow.org) which is based on R scripts. Two-way ANOVA with FDR correction was performed to highlight line status or water-treatment effects and interaction. The parameters used for partial least squares-discriminant analysis (PLS-DA) in BioStatFlow were adjusted to a tenfold cross-validation (CV) to generate the model (and calculate the Q^2^) and 200-randomized permutations to estimate the robustness of the generated model. Some graphical outputs for PLS-DA were produced by mixOmics, using the same parameters than with BioStatFlow. An additional R script from Fu et al. (2017) was used to perform least absolute shrinkage and selection operator (LASSO) and sparse partial least square (sPLS) selection. Principal component analysis (PCA) and partial least square (PLS) were performed on data mean-centred and scaled to unit variance. All statistical analyses were performed on the data set in Online Resource 2 or subsets of this file.

## Results

### Sunflower leaf metabolic contents measured by targeted and untargeted approaches

**Fig. 1:**
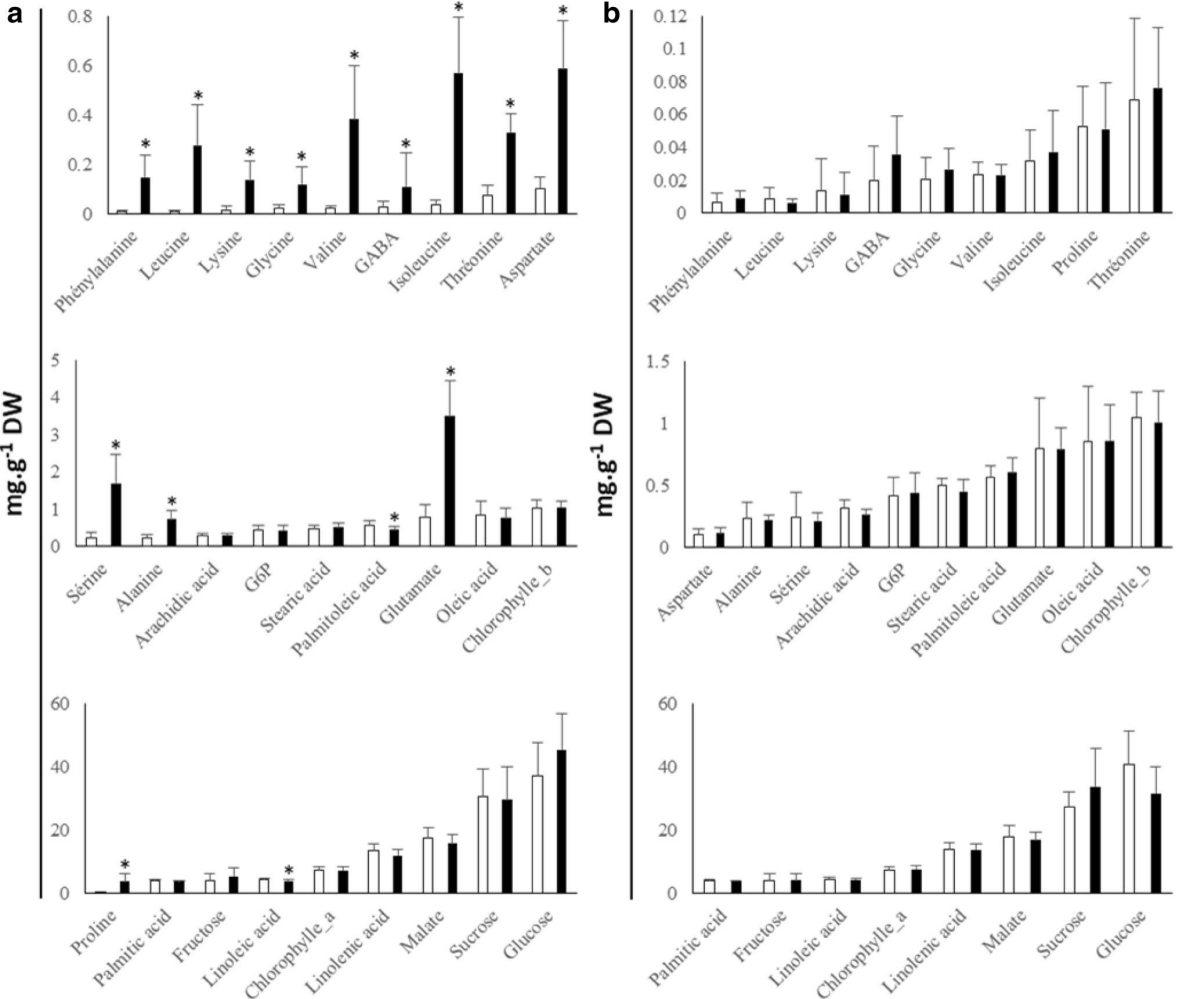
Concentrations of 27 metabolites measured by targeted methods (UPLC-Fluo for amino acids, GC-FID for FAMES, spectrophotometry for others) in leaf of B or R sunflower lines cultivated in two conditions (WW and DS). Results are expressed in mg g^−1^ DW in the four types of samples. **a** WW (white bars) or DS (black bars). **b** Maintainer B lines (white bars) or restorer R lines (black bars). Vertical bars represent standard deviations. Asterisk indicates variables that were found significantly different between groups after two-way ANOVA test (*p* value < 0.05)

In total, 27 metabolites plus starch and protein content were targeted and quantified in sunflower leaf. Major soluble sugars (i.e. the ones with the highest content), organic acids and chlorophylls were quantified with spectrophotometric analyses. FAMES and free amino acids were measured by using GC-FID and UPLC-fluo, respectively. These data are presented for the different conditions in Fig. 1 and Online Resource 3—Fig. S1. We targeted these compounds because they are (1) often considered as putative metabolic markers (Fernandez et al. 2016) and (2) valuable candidates for a high-throughput metabolic marker approach, as they are easy and cheap to measure.

The concentrations of these 29 compounds were summed to estimate their contribution to leaf biomass. This yielded about 45% of leaf dry mass. Glucose was found to be the major soluble sugar. Its concentration (32–45 mg g^−1^ DW) was in the same range as that of sucrose, but 8–10 times higher than fructose depending on the chosen conditions. Glutamate, alanine and serine were found to be the most abundant amino acids. In leaves, linolenic acid (C18:3) was the most abundant fatty acid (7.5–18.6 mg g^−1^ DW), followed by linoleic acid (Fig. 1).

^1^H-NMR profiling was performed on polar extracts to further analyse metabolites from primary metabolism in the millimolar range. Four hundred and seventy-nine regions were observed in the ^1^H-NMR cpmg dataset, of which 20 compounds were annotated (Online Resource 4). Eleven identified compounds were measured and quantified with the ^1^H-NMR quantitative single-pulse dataset, but only nine of them were kept in the final dataset to avoid redundancy with targeted spectrophotometric measurements. When summed, these compounds represented an additional 5% of the leaf dry mass (Online Resource 4).

LC–ESI–QTOF–MS analysis of semi-polar extracts was performed to analyse specialized metabolites. The most intense peaks that were detected in the sample extracts, based on their intensity in the XCMS table generated by a relative area under curve (AUC) approach, were tentatively annotated. Orbitrap-MS data were used in order to gain precision on mass measurement and to perform MS/MS. Online Resource 5 shows the annotation table generated using a representative spectrum of a leaf extract with annotation of the most intense peaks. The two most intense peaks were annotated as mono and di-caffeoyl quinic acid. With a retention time around 17–20 min, several methylated flavonoids were also detected. Finally, three smaller peaks ranging in retention time from 15 to 17 min were found to putatively represent sunflower sesquiterpenoids. Several peaks after 25 min remained elusive.

Several metabolite concentrations differed between the conditions, as highlighted by a two-way ANOVA (*p* < 0.05 with FDR correction, Online Resource 6—Table S1a).

#### Difference between DS and WW samples

The most striking difference was the large increase in each individual amino acid concentration found for DS samples, with an average increase of 15-fold, (Fig. 1a, Online Resource 6—Table S1a). On the other hand, starch, protein content, linolenic and palmitoleic acids were slightly but significantly lower in DS (Fig. 1a; Online Resources 3—Fig S1a and 6—Table S1a). Minor differences in starch, protein, soluble sugars and GABA were observed between B and R lines (Fig. 1b and Online Resource 3—Fig S1b) but none of them was statistically significant (Online Resource 6—Table S1a).

Among the variables that were highly significant under DS (two-way ANOVA test), most of them were unidentified ^1^H-NMR spectra regions (Online Resource 6—Table S1a). Among them, myo-inositol, glycine betaine and trigonelline were significantly higher under DS, whereas chlorogenate and formate were significantly lower (two-way ANOVA test; Online Resources 4 and 6—Tables S1a). For the other nine compounds identified in ^1^H-NMR spectra (amino acids and sugars), excellent correlations were found with spectrophotometric and chromatographic targeted methods (data not shown).

Finally, only a small group of m/z were significantly different under DS (Online Resource 6—Table S1a). Four of them were putatively annotated as heliannuol, 3-*O*-caffeoylquinic acid, tryptophan and phenylalanine. The last two were also detected by the UPLC-fluo targeted method.

#### Difference between B and R samples

For the B and R lines, no targeted metabolites were significantly different. Two unidentified ^1^H-NMR variables had a *p* value < 0.05 (Unk_6.8936 and Unk_3.8733, Online Resource 6—Table S1a,). However, except for chlorogenic acid, most organic acids measured displayed a lower concentration in R lines leaf samples (Fig. 1b, Online Resource 4). Finally, the rest of the variables that were found significantly different for line status were unidentified MS-based variables (Online Resource 6—Table S1a), except for two putatively annotated flavonoids (Online Resources 5 and 6—Table S1a).

### Workflow for identifying metabolic markers of water treatment and line status

**Fig. 2:**
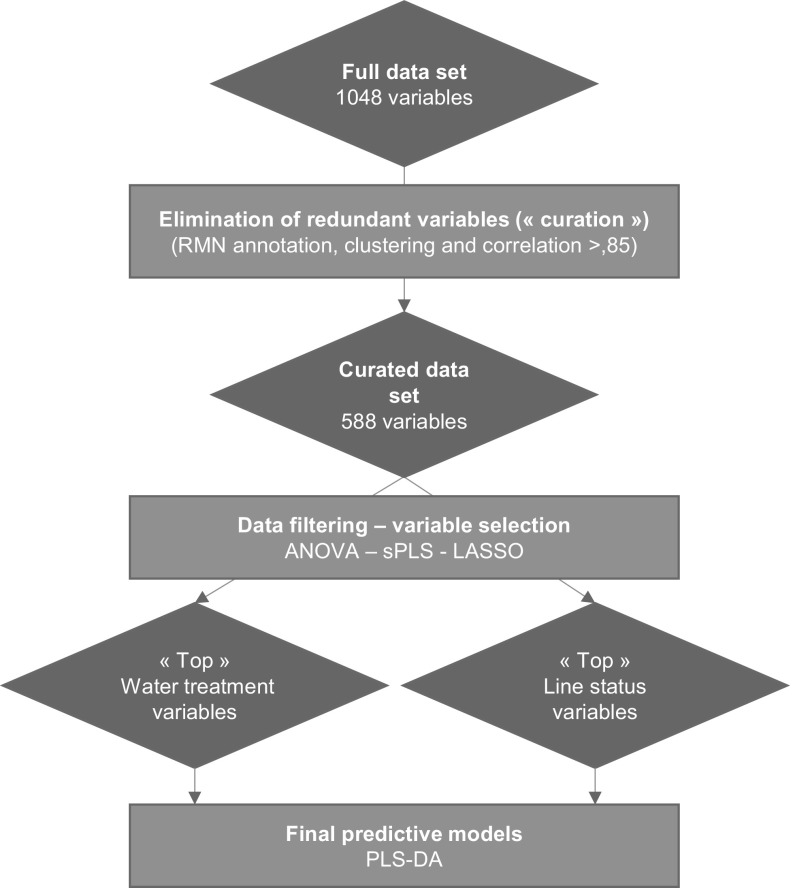
Description of the statistical analysis pipeline used in this article

The analytical methods allowed the generation of a matrix of 1048 metabolic variables (Online Resource 2). This matrix included 27 targeted metabolites, starch, total protein content and 9 annotated ^1^H-NMR variables. The remaining variables were composed of ^1^H-NMR unidentified spectral regions and 540 MS-based signatures. The matrix was processed through a three-step biostatistical pipeline to select the more relevant variables to discriminate samples according to water treatment and line status: (1) elimination of redundant variables, (2) variable selection for each sample cluster and (3) final PLS-DA model calculation (Fig. 2).

#### Elimination of redundant metabolic variables

Since a single metabolite can be encompassed within several ^1^H-NMR buckets or MS-based ions, we first reduced this full data set by hierarchical clustering (BioStatFlow, Pearson correlation, average linkage as aggregation method). Clusters were generated with a correlation threshold of 0.85. Within each cluster, MS-based metabolic variables corresponding to adducts or isotopes were eliminated while the one with the highest AUC was kept. For ^1^H-NMR buckets, we used a similar process in order to keep buckets bearing the highest AUC. After this curation process (Fig. 2), the new dataset comprised 588 variables (Online Resource 7).

**Fig. 3:**
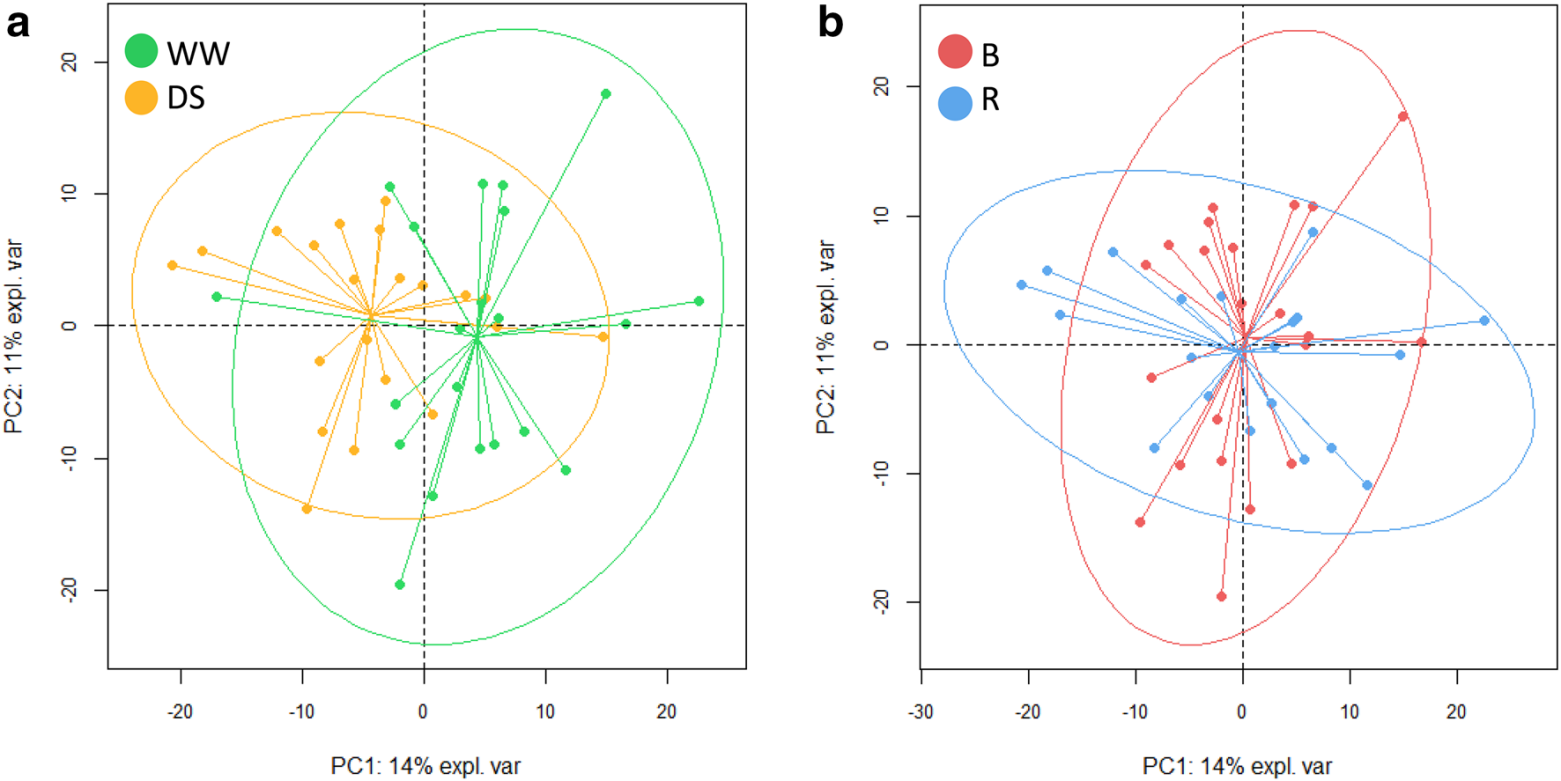
PCA scores plot (PC1 x PC2 plan) generated with the full set of 588 metabolic variables (Online Resource 7) measured in sunflower leaf cultivated in a Heliaphen phenotyping platform. **a** Highlighting samples with different water treatment. WW, green dots and DS, orange dots. **b** Highlighting line types. B, red dots and R, blue dots. Coloured ellipses represent 95% confidence level. The connecting lines attach each individual point to the centre of the confidence ellipse

We then tested the discrimination potential of this curated data set on our sample groups using an unsupervised statistical approach. PCA was first carried out (Fig. 3). The first two components displayed in Fig. 3a (water treatment) and Fig. 3b (line status) explained 25% of the total variability. The separation of our sample groups was incomplete, although slightly better for DS. We then performed a supervised method (PLS-DA) on this 588-variable dataset for each type of sample group. Each PLS-DA analysis was able to discriminate WW from DS samples (Online Resource 3—Fig. S2a), and B from R lines (Online Resource 3—Fig. S2b), in the 2D space based on the first two latent variables. Predictive ability (Q^2^) and proportion of variance (R^2^) explained by the model were higher than 0.9 and 0.8 in both cases (Table 1), respectively. Each model was considered as valid as it bore Q^2^ and R^2^ values above 0.4 and 0.5, respectively (Patil et al. 2016). However, in a high-throughput approach, it is impractical to measure more than 500 variables to discriminate or predict cluster differentiation. Therefore, our next step was to test a variable selection process and to assess the validity of group discrimination with PLS-DA after this selection. PLS-DA was chosen to easily compare model performance using Q^2^ values.

**Table 1:** Comparison of predictive ability (Q^2^) and explained variance explained (R^2^) of the different PLS-DA models calculated with different selected data sets

Variable selection	Condition	Data set size	Q^2^	R^2^ Expl var t1/year (%)	CV p-value
None	Water treatment	588 Variables	0.936	80.2	1.1E−04
	Line status	588 Variables	0.916	89	3E−04
ANOVA	Water treatment	90 Variables	0.964	83.70	3.04E−03
		50 Variables	0.96	88.6	9.00E−05
		20 Variables	0.974	83.7	2.71E−03
	Line status	35 Variables	0.911	75.60	1.12E−03
		20 Variables	0.9	76.10	9.00E−05
LASSO	Water treatment	90 Variables	0.982	88.90	1.47E−03
		50 Variables	0.982	93.1	2.60E−04
		20 Variables	0.985	88.90	1.47E−03
	Line status	35 Variables	0.973	92	3.29E−03
		**20 Variables**	**0.978**	94.30	6.00E−05
sPLS	Water treatment	90 Variables	0.985	92.90	8.90E−03
		**50 Variables**	**0.992**	96.40	6.00E−04
		20 Variables	0.988	92.90	4.90E−03
	Line status	35 Variables	0.97	82.30	1.36E−03
		20 Variables	0.934	79.60	5.00E−04
Custom	Water treatment	8 Variables Metabolites	0.96	85.9	6.00E−05
		6 Variables Physiological	0.686	53.9	3.00E−05

Variable selection conditions, cluster and the number of variables used are indicated. Permutation robustness was assessed with 200 CV cycles. The data set providing highest Q^2^ was highlighted in bold font

#### Metabolic variable selection process

To select variables, we compared three different methods for each condition (DS or line status), a generalised univariate method (one-way ANOVA) and two multivariate ones (sPLS and LASSO penalty; Fu et al. (2017); Fig. 2). The 588-variable data matrix (Online Resource 7) was submitted to these methods and subsequent PLS-DAs were performed. We compared the Q^2^ and R^2^ to assess the quality of the variable selection process for each resulting PLS-DA model (Table 1). Since our objective was to find the smallest possible variable set, we analysed datasets of different sizes (90, 50 and 20 variables for water treatment; 35 and 20 variables for line status). We dimensioned the first selected data set size according to the numbers of variables with a *p* value < 0.05 following one-way ANOVA (90 for DS and 35 for line status). We then reduced the data set size down to 20, a reasonable number of metabolic variables to measure when using metabolic markers in a high-throughput manner (see discussions on practicality of metabolic markers in Fernandez et al. 2016). For DS, we chose to add an intermediate data set of 50 variables. Q^2^, R^2^ and CV-*P*-values of individual models are summarized in Table 1.

**Fig. 4:**
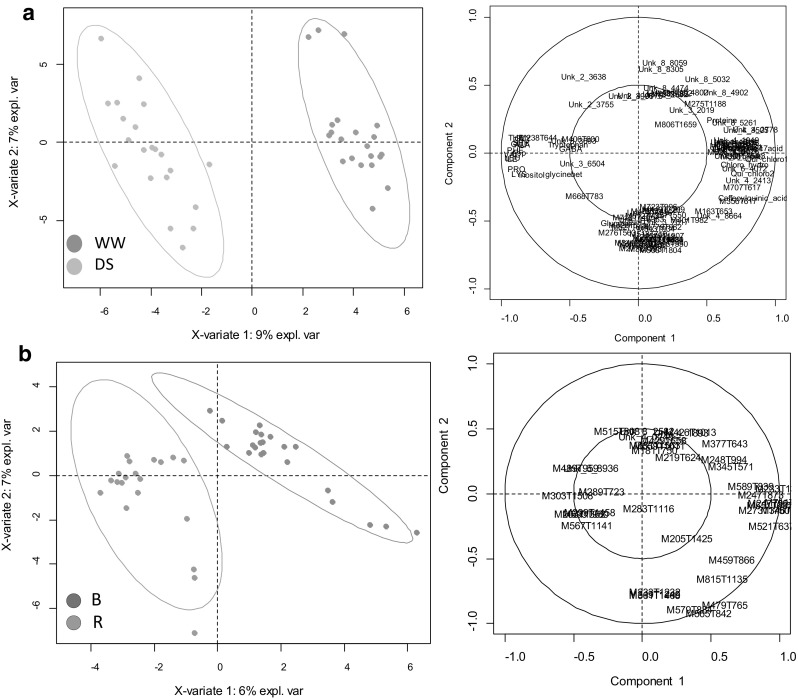
PLS-DA of metabolic data sets of sunflower leaf on variables selected from the set of 588 metabolic variables (Online Resource 7) after a selection process based on sPLS or LASSO. **a** PLS model scores (left) and loadings plot (right) of the 50 best sPLS selected variables discriminating the two water treatments WW (green dots) and DS (orange dots). **b** PLS model scores (left) and loadings plot (right) of the 20 best LASSO selected variables discriminating the two-line types, B maintainer lines (red dots) and R restorer lines (blue dots). Coloured ellipses represent 95% confidence level

The randomized permutations for validation200 cycles) of each bore a significant *p* value, thus demonstrating their robustness (Table 1). As expected, the resulting models computed after the selection process displayed a higher Q^2^ when compared to the previous PLS-DA performed with 588 variables (Table 1; Online Resource 3—Fig. S2). The ANOVA selection process produced efficient models but with the lowest Q^2^ in all situations (Table 1). sPLS and LASSO selection resulted in more discriminant models, the latter for line status and the former for water treatment. The most efficient PLS-DA models are illustrated in Fig. 4: 50 variables for water treatment (sPLS selection) and 20 variables for line status (LASSO selection) as well as PCA computed with the same data sets (Online Resource 3—Fig. S3).

#### Metabolic VIP analyses

In PLS-DA, an important feature is the variable importance in the projection (VIP) scores. High VIP-score variables strongly contribute to the PLS-DA model. Variables with VIP scores higher than 1 are listed in Online Resource 6. No matter which variable selection process was applied, amino acids were overrepresented in the high VIP-score shortlist, underlying their importance in discriminating DS and WW samples in our experiment (Online Resource 6—Table S1b). Two other variables measured by ^1^H-NMR were listed in the VIPs shortlist in nearly all conditions of variable selection: inositol and glycine-betaine (Online Resource 6—Table S1b). On the other hand, a small number of LC–MS-based variables had VIP scores higher than 1 (Online Resource 6—Table S1b). For line status discrimination, all variables with VIP scores higher than 1 were unidentified ions or ^1^H-NMR spectral regions (Online Resource 6—Table S1c).

### Cost-efficient metabolic markers

Simplicity of measurement and cost-efficiency of metabolic markers are arguably as important as their prediction capacity (Fernandez et al. 2016). In other words, measuring a set of markers with a (slightly) lower predictive capacity might be relevant if the marker set is easier or cheaper to measure. A simple solution is often to replace untargeted methods with targeted ones. We estimated the cost-reduction potential by a factor of 3–20 (Fernandez et al. 2016). Another possibility is to measure globally a family of compounds when they are affected in the same way by a given treatment or condition, like in our case for amino acids in DS samples (Fig. 1a).

**Fig. 5:**
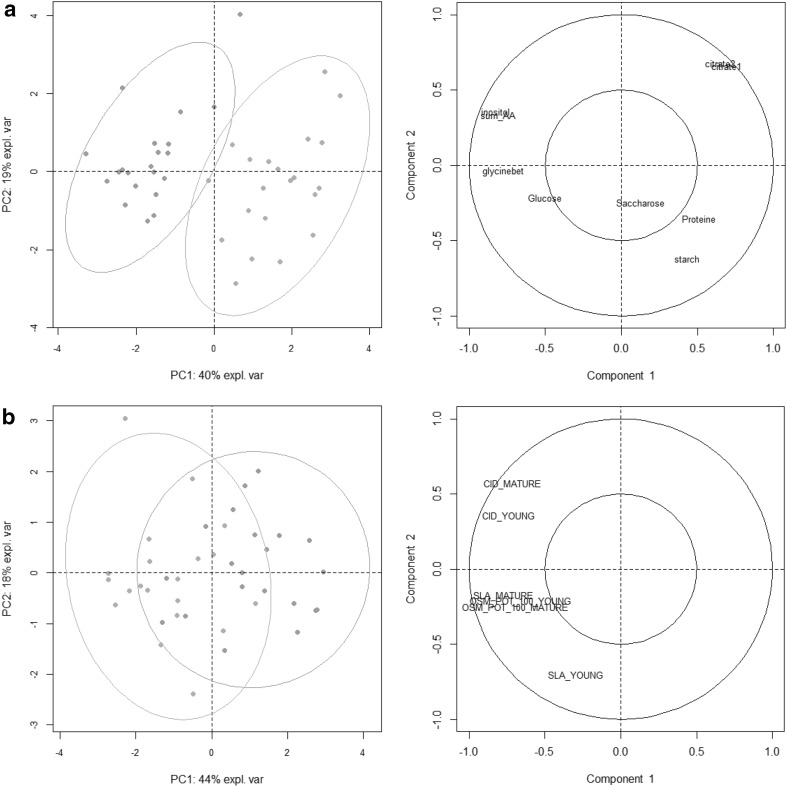
PCA scores plot generated with **a** an “easy-to-measure” data set (total free amino acids, citrate, glycine-betaine, inositol, glucose, total proteins and starch) and **b** six physiological variables (SLA, OSM_POT and CID) measured the day before final sampling. Left, scores plot. Right, loadings plot

To illustrate this point, we selected metabolic variables (from Online Resource 7) known to be simple or cheap to measure and relevant for water treatment discrimination. Since all free amino acids measured were increased in DS samples, we replaced them by a single variable representing their sum (hereafter called total free amino acids). Finally, we chose total free amino acids, citrate, glycine-betaine, inositol, sucrose, glucose, protein and starch. This set of eight variables was offered a clear determination of DS and WW samples in an unsupervised analysis (PCA, Fig. 5a). Additionally, the generated PLS-DA model was efficient with Q^2^ = 0.96, and R^2^ = 0.55 (Table 1, Online Resource 3—Fig S4a). We could not perform this approach for line status since most of their high VIP-score variables were unidentified metabolic signatures.

### Comparison with physiological variables for DS markers

Physiological markers are used to assess the impact of DS on plant. In our experiment, SLA, OSM_POT and CID were measured in young and mature leaves at the end of DS. To test the quality of our PLS-DA model built with selected metabolic variables, we compared its discriminative capacity with a PLS-DA model built with this physiological dataset comprising six variables extracted from a larger dataset published in Blanchet et al. (2018). Unsupervised PCA computed with this dataset showed poor separation of DS and WW samples (Fig. 5b). Furthermore, the PLS-DA model built with these physiological data displayed a Q^2^ = 0.68 and an R^2^ = 0.54 (Table 1, Fig. 4b), but was less efficient than those built with the minimal set of eight metabolic variables (Q^2^ = 0.96, R^2^ = 0.55; Table 1, Online Resource 3—Fig S4a).

## Discussion

### Sunflower leaf metabolite composition

Sunflower is an important crop that provides most of the table oil used worldwide. However, few metabolomic data are available to date concerning both its primary and specialized metabolism. We now present one of the largest sets of primary metabolites in adult sunflower leaf, with absolute quantification of 38 metabolites and with several compounds not quantified by Moschen et al. (2017) using GC–MS.

Several points can be made about sunflower leaf composition. Malate, citrate and chlorogenic acid were the major organic acids (Fig. 1, Online Resource 4) and linolenic acid, linoleic acid and palmitic acid were the major fatty acids detected. This is in contrast with the fatty acids in sunflower seed where linoleic acid is the most abundant. Serine, alanine and glutamate were the major free amino acids (Fig. 1). Glucose and sucrose were the major soluble sugars in leaf but their concentrations were at least eight times higher than that of fructose. This might be due to some specificity of the fructose metabolism in the Asteraceae family. In sunflower, fructose is not metabolized into inulin (a fructose-derived polymer) but is transported and then accumulated in the stem. For example, Martínez-Noël et al. (2015) found that fructose was three times more concentrated than any other soluble sugar in this organ. This might explain the difference between glucose and fructose concentrations in our leaf samples.

Considering the specialized metabolites detected via LC–ESI–QTOF–MS, the peaks presenting the highest intensities were putatively annotated (Online Resource 5). They include compounds from three families: caffeoylquinates, methyl-flavonoids and sesquiterpenoids. These compounds had all been previously detected in sunflower biochemical analyses. Caffeoylquinic acid is a compound commonly found in sunflower. It plays a role in lignification and correlates with leaf age in sunflower (Koeppe et al. 1970). It is the dominant phenolic acid in sunflower florets (Liang et al. 2013) and is also present in seeds (Karamać et al. 2012; Pedrosa et al. 2000). When present in sunflower oil, caffeoylquinates including oxidized chlorogenic acid can generate green-coloured oxidized complexes by reacting with sunflower proteins (Wildermuth et al. 2016). This oxidative reaction between chlorogenic acid and proteins partly explains why sunflower proteins are still underused in the food industry, despite their qualities such as their cheapness and absence of allergens (Wildermuth et al. 2016). Several putative methylated flavonoids were also detected (Online Resource 5). These compounds have been used as chemotaxonomic markers for the Astereaceae family (Emerenciano et al. 2001). Finally, specific sunflower sesquiterpenoids were also detected, one of which was putatively identified as niveusin. In sunflower, this compound and its derivatives are thought to offer potential as insecticides (Prasifka et al. 2015).

### Variable selection process

Variable selection is necessary in metabolomics, especially when looking for metabolic markers (Fernandez et al. 2016). However, numerous methods can be used for the variable selection process and have already been the subject of discussion (for review, Grissa et al. 2016). We submitted our initial dataset to three variable selection processes: ANOVA, sPLS and LASSO penalty.

### Biomarkers of line status

Leaf samples of R and B lines were discriminated with the metabolic data set mostly through unidentified markers measured by LC–ESI–QTOF–MS (Online Resource 6—Table S1c). R lines, which in sunflower breeding are used to restore the CMS phenotype, have a nuclear-encoded *Rfl* gene that might act as a transcriptional activator (Balk and Leaver 2001; Chen and Liu 2014). The only known function of the *Rfl* gene is to restore male fertility in CMS plants (Chen and Liu 2014) as well as the associated changes restricted to the mitochondria of floral tissues linked with this loss of fertility (i.e. mitochondrial membrane integrity and respiration ratio). Phenotypes associated with the presence of *CMS* or *R* genes are thought to be limited to floral tissues. The fact that we were able to discriminate R and B lines using analyses of leaf metabolites suggests that the phenotype is not restricted to flowers and that it might affect other plant tissues and organs. Interestingly, several organic acids were less concentrated in R line samples, although not individually significantly. This might be due to an effect on the mitochondrial metabolism in all organs, but this hypothesis needs to be confirmed. Further annotations of the associated markers would contribute to propose hypotheses about direct or indirect *R* gene effects in leaf. Additionally, metabolomic markers denote intermediate information between genes and final phenotypes and might capture multilocus-controlled traits and associate alleles producing the same final phenotype. The latter property would be interesting in breeding programs to predict the restoration phenotype of novel alleles in pre-breeding programs and therefore to identify novel sources of restoration for the PET1. However, further biochemical and statistical analyses with more R and B lines are required since PLS-DA may be prone to overfitting.

### Biomarkers of water treatment

The discrimination of WW and DS samples using metabolic variables was more efficient than the discrimination of line status. Amino acids were clearly the best DS markers in our dataset, displaying a 5- to 10-fold increase in DS sunflower leaves (Fig. 1a). Increases in amino acids under DS in sunflower have already been documented, although to a lesser extent and in a cultivar-dependent manner (Manivannan et al. 2007). This feature has also been detected in other crops such as barley (Lanzinger et al. 2015) and wheat (Bowne et al. 2012). Conversely, Moschen et al. (2017) found that the concentrations of several leaf amino acids were decreased under DS in sunflower (Correia et al. 2005). These contradictory results regarding amino acid responses might be due to water–stress intensity, sampling stage or differences in nitrogen nutrition. In the present study, the use of Heliaphen high-throughput phenotyping platform allowed the application of a precise and reproducible drought scenario that is available for more thorough understanding of the impact of DS on leaf metabolism. Nevertheless, higher concentrations of individual amino acids such as proline and glycine have been detected in DS leaves (Moschen et al. 2017). Amino acids, and especially proline, might participate in osmotolerance under DS, although the case is highly debated for the latter (Szabados and Savouré 2010).

In our dataset, other metabolites appeared as good markers of DS samples, i.e. glycine-betaine and myo-inositol. Glycine-betaine is accumulated in various plants under abiotic stress (Giri 2011). Generally, plants accumulate amounts of glycine-betaine that are too low to significantly impact the sap osmotic potential. Rather, it might serve as a ROS detoxication agent (Giri 2011). In the case of myo-inositol, Taji et al. (2006) suggested it might be involved in osmotolerance, or alternatively serve as a secondary messenger involved in phospholipid signalling pathways. Finally, caffeoylquinates and sesquiterpenoids (a terpene class with three isoprene units) were also detected as putative markers of DS versus WW samples (Online Resources 5 and 6). Caffeoylquinates have been associated with DS responses in grapevine (Hochberg et al. 2013). Terpenes have been shown to be involved in thermotolerance and antioxidant effects (Sharkey et al. 2008). Furthermore, terpenes seem to have radical scavenging activity contributing to the mitigation of oxidative damage during stresses. In sunflower leaf, genes involved in terpene metabolism have been shown to be upregulated under drought conditions (Moschen et al. 2017).

### Towards a small efficient biomarker dataset

Fernandez et al. (2016) argued that ideal metabolic markers should be easy and cheap to analyse. For this purpose, we tested the discriminant capacity of a small metabolic marker set composed of eight biochemical variables: total free amino acids, citrate, glycine-betaine, myo-inositol, sucrose, glucose, total proteins and starch. An unsupervised PCA clearly separated WW and DS samples when these eight biochemical variables were used (Fig. 4a), but not with the physiological dataset consisting in six common indicators of DS measured at plant level. Indeed, SLA, OSM_POT and CID (measured in both young and mature leaves) are often used to characterise the water–stress status of a given crop (Fig. 4b). This was confirmed when comparing Q^2^ values for PLS-DA models computed with each of these data sets (0.91 and 0.68 respectively). However, since amino acids were overrepresented in our PLS-DA model VIPs, our approach might not be generalizable to any given criterion. Indeed, reducing the number of variables was much less efficient in discriminating line status. Furthermore, given the fact that amino acid accumulation is not always reported for sunflower experiencing drought, more studies with various drought scenarios and more lines will be required to confirm our conclusions. Finding the right balance between cost reduction and prediction efficiency of each metabolic marker set is likely an achievable goal in many situations but will certainly require optimisation for each performance criterion studied.

## Conclusions

Metabolic markers are a recent development in science. Applications such as personalized medicine have recently attracted keen interest (Lindon and Nicholson 2014). Their use in agronomy as a potential tool for crop breeding is even more recent (Fernandez et al. 2016). In the present work, we show that a limited number of metabolic markers can discriminate plant sample groups with different characteristics or treatment applications, especially in the case of DS. This feature was already noted at early stages of plant development in maize (Riedelsheimer et al. 2012). The fact that leaves of sunflower lines carrying different alleles of the CMS restoration gene were separated by this approach shows that metabolomics can reveal an unsuspected metabolic phenotype in a given organ. The present work also emphasizes the importance of variable selection. The pipeline we propose (Fig. 2) may not be optimised for all situations (sample numbers, organ types, analytical approaches…), but will provide a preliminary guideline for future users. Another important point is the specificity of the list of selected markers towards the selected stress. Indeed, several metabolites could be considered as valid metabolic markers of different stresses, simply because their concentrations may be significantly altered under various stress situations. To alleviate this bias, these markers should be tested under various stress scenarios (Fernandez et al. 2016). It will indeed be crucial to verify whether such a modelling approach remains valid when the predictive metabolomic data and the predicted phenotypic data are obtained in different experiments. In particular, the possibility to use young plantlets, grown in controlled conditions and “metabotyped”, to predict a field phenotype of interest could be extremely useful especially regarding cost reduction, but will require extreme caution. Thus, a careful methodology with a clear choice of performance criteria (see Fernandez et al. 2016), stress scenario, developmental stages and analytical methods will have to be developed to test this hypothesis.

## Electronic supplementary material

Below is the link to the electronic supplementary material.

Supplementary material 1 (DOCX 26 kb)

Supplementary material 2 (XLSX 551 kb)

Supplementary material 3 (PPTX 239 kb)

Supplementary material 4 (XLSX 89 kb)

Supplementary material 5 (DOCX 30 kb)

Supplementary material 6 (XLSX 76 kb)

Supplementary material 7 (XLSX 325 kb)

## Abbreviations

AQC: 6-Aminoquinolyl-*N*-succinimidyl carbamate
AUC: Area under curve
B: Maintainer line
CID: Carbon isotope discrimination
CV: Cross-validation
DAG: Days after germination
DS: Drought stressed
DW: Dry weight
FAMES: Fatty acid methyl esters
LASSO: Least absolute shrinkage and selection operator
LC–ESI–QTOF–MS: Liquid chromatography–electrospray-ionization–time-of-flight–mass spectrometry
NMR: Nuclear magnetic resonance
OSM_POT: Osmotic potential
PCA: Principal component analysis
PET: Petiolaris
PLS: Partial least squares
PLS-DA: Partial least squares discriminant analysis
R: Restorer line
SLA: Specific leaf area
VIP: Variable importance in the projection
WW: Well-watered
