# Supplementary material for: Metabolomic characterization of sunflower leaf allows discriminating genotype groups or stress levels with a minimal set of metabolic markers
Source: Metabolomics. 2019 Mar 30;15(4):56. doi: 10.1007/s11306-019-1515-4 (PMC6441456; doi:10.1007/s11306-019-1515-4)
Supplement: Supplementary file 1 — Supplementary material 1 (DOCX 26 kb) [file 11306_2019_1515_MOESM1_ESM.docx]

**Title**: Metabolomic characterization of sunflower leaf allows discriminating genotype groups or stress levels with a minimal set of metabolic markers

**Authors**: Olivier Fernandez, Maria Urrutia, Thierry Berton, Stéphane Bernillon, Catherine Deborde, Daniel Jacob, Mickaël Maucourt, Pierre Maury, Harold Duruflé, Yves Gibon, Nicolas B. Langlade, Annick Moing

**Journal**: Metabolomics

**Online Resource 1:** Supplementary materials and methods

***Plant material and growth conditions***

The experiment was performed from May to July 2013 in the outdoor high-throughput phenotyping platform “Heliaphen” as previously described in (Gosseau et al. 2018). Detailed protocol is presented in (Blanchet et al. 2018) data paper.

Sterilized seeds were germinated on Petri dishes and then plantlets were transplanted into individual pots (Blanchet et al. 2018). Eight sunflower lines, four B maintainers (SF009, SF092, SF109, SF193) and four R restorers (SF279, SF279, SF317, SF342) were grown in two conditions (Well-Watered; WW or Drought-Stressed; DS) with three replicates per line arranged in a split-split-plot design with three blocks. Plants were part of a larger experimental design also including hybrids not studied here (Badouin et al. 2017). Pairs of DS and WW plants were harvested when the fraction of soil water available (FTSW) of the DS plants reached 0.1 (occurring between 42 and 47 DAG). For each plant, mature and young leaves were harvested from 11:00 to 13:00 for metabolomic analyses. They were cut without their petiole and frozen immediately in liquid nitrogen, and two other leaves (mature and young leaves) were cut for physiological trait measurements. During the experiment, two samples were excluded before leaf sampling (excessive irrigation was detected when analysing final Heliaphen readings) and four samples could not be analysed because of insufficient powder quantity. This resulted in a total of 42 samples submitted to metabolic analyses.

***Physiological trait measurements for plant phenotyping***

Plant and leaf physiological data are part of a larger dataset presented in Blanchet et al. (2018). Specific leaf area (SLA) was determined with leaf discs (3.1 cm diameter) cut on rehydrated lamina and dried (48 h, 80°C), and expressed in m^2^.kg^-1^ (Allinne et al., 2009). Both leaf osmotic potential (OSM_POT) and leaf osmotic potential at full turgor (OSM_POT_100) were measured on expressed sap of frozen and thawed leaves using 10 ml aliquots placed in an osmometer (Wescor Model 5520, Logan, Utah, USA), as described in (Poormohammad Kiani et al., 2007).

To assess Carbon isotope discrimination (CID), the samples of oven-dried leaves used for SLA measurements were ground. A subsample of 3 mg was weighed and placed in capsules (Elemental Microanalysis, Okehampton, UK) and analysed using a continuous low isotope ratio MS at the Stable Isotope Platform SHIVA (University of Toulouse, France).

***Targeted compound measurements***

For each sample, about 20 mg fresh weight were extracted as by (Hendriks et al. 2003). Sucrose, glucose, and fructose (Jelitto et al. 1992), malate (Nunes‐Nesi et al. 2007), citrate (Tompkins and Toffaletti 1982) and glucose-6-P (Gibon et al. 2002) were determined in the ethanolic supernatant. Starch (Hendriks et al. 2003) and protein (Bradford 1976) contents were determined on the pellet resuspended in 100 mM NaOH. Assays were prepared in 96-well microplates using Starlet pipetting robots (Hamilton, Villebon sur Yvette, France), and absorbance was read at 340, 570, or 595 nm in MP96 microplate readers (SAFAS, Monaco, Monaco).

Individual free amino acids analysis was carried out using an UPLC separation with a fluorescent detection after derivatization using 6-aminoquinolyl-*N*-succinimidyl carbamate (AQC)-tag. Derivatization was performed using the AccQ-Fluor reagent kit (Waters, Saint-Quentin-en-Yvelines, France) according to the manufacturer’s instructions. Amino acid derivatives were separated on an Acquity BEH C18 column (2.1 × 100 mm, 1.7 µm; Waters). Solvents were AccQ-tag Ultra eluent A (Waters) and acetonitrile/water (25 v:75 v). Detection was performed at 473 nm after excitation at 266 nm. Quantities of individual amino acids were calculated with external calibration curves prepared from commercial amino acid standards (Sigma, Saint-Quentin Fallavier, France).

For lipid analysis, fatty acid methyl esters (FAMES) were measured after hydrolysis of 20 mg DW with 2.5% H_2_SO_4_ (v/v) in methanol (and 1 µg/ml heptadecanoic acid as internal standard). Tubes were heated at 80 °C for 1 h and cooled to room temperature. Four hundred µl of hexane and 1.5 ml of H_2_O were added to extract FAMES. Tubes were shaken vigorously and centrifuged, and organic phases were transferred into injection vials. GC-FID was performed using an Agilent 7890 gas chromatograph (Santa Clara, California) equipped with a Carbowax column (15 m x 0.53 mm, 1.2 µm; Alltech Associates, Deerfield, IL, USA) and flame ionization detection. The chromatography temperature gradient was 160°C for 1 min, increase to 190°C at 20°C/min, then to 210°C at 5°C/min, and finally 210°C for 5 min. FAMES were identified by comparing their retention times with commercial fatty acid standards (Sigma, Saint-Quentin Fallavier, France) and quantified using ChemStation Agilent, Santa Clara, California) to calculate the peak areas and compare them with the C17:0 response.

***^1^H-NMR analysis of major polar compounds***

Polar metabolites were extracted on lyophilized powder (40 mg DW per biological replicate) with an ethanol–water series (80/20, 50/50, 0/100 v/v) at 80 °C as described in (Deborde et al. 2009) with minor modifications. This three-step extraction process (ethanol-water series) was chosen to take into account the diverse affinities and solubilities of leaf major polar compounds (i.e. sugars, organic acids, amino-acids) for ethanol or water, in order to obtain an accurate view of these compounds in leaf extracts. The lyophilized extracts were solubilized in deuterated phosphate solution (100 mM, apparent pH 6.0) and were adjusted to apparent pH 6.00±0.02 using NaOD or DCl by means of a titration robot (BTpH, Bruker, Kalrsruhe, Germany) and lyophilized again. Each dried pH-adjusted extract was solubilized in 0.5 ml of D_2_O with (trimethylsilyl) propionic-2,2,3,3-d4 acid (TSP) sodium salt (0.01% final concentration) for chemical shift calibration and EDTA.Na_2_ (2 mM final concentration). The mixture was centrifuged at 10,000 *g* for 5 min at room temperature. The supernatant was then transferred into a 5 mm NMR tube (Wilmad, Vineland, USA) for NMR acquisition. ^1^H-NMR spectra for fingerprinting or profiling were recorded at 500.162 MHz on a Bruker Avance III spectrometer (Bruker, Karlsruhe, Germany) using an ATMA inverse 5-mm probe flushed with nitrogen gas, at 300 K. Two pulse sequences were used, a CPMG sequence for resolution purpose for fingerprinting, and a zg single-pulse sequence for quantitative purpose for profiling. In order to get the same constant temperature of the sample set run, a delay (90 s) for temperature homogenization of the sample in the magnet was used. To optimize NMR conditions, automated tuning and matching, locking, shimming (topshim) and 90° hard pulse calibration (pulsecal) were carried out for each sample. For each pulse sequence, the same dedicated receiver gain was used for all samples. For NMR fingerprinting, thirty-two scans of 64k data points each were acquired with a CPMG sequence (cpmgpr1d), a 90 ° pulse angle, a 6000 Hz spectral width, a 5.45 s acquisition time, a 5 s recycle delay, 500 µs echo time and 150 loops, preceded by16 dummy scans. For NMR quantitative profiling, sixty-four scans of 32k data points each were acquired with a single-pulse sequence (zg), a 90 ° pulse angle, a 6000 Hz spectral width, a 2.73 s acquisition time, and a 15 s recycle delay.

Apodisation (LB 0.3Hz), zero-filling (X2) and Fourier transformation of Free Induction Decay, phasing, chemical shift calibration and baseline correction of spectra, peak realignment, non-uniform bucketing and signal-over-noise ratio determination were carried out with the NMRProcFlow web tool (((Jacob et al. 2017) www.nmrprocflow. org)). Each spectral region of interest or bucket was determined manually with either the intelligent bucketing module or variable size bucketing module of NMRProcFlow for each data set. Each bucket is designated according to the central chemical shift value, in ppm, of the spectral region. This resulted, for cpmg dataset, into 479 normalized (constant sum normalization) variables or buckets for cpmg dataset for fingerprinting and 20 regions of interest for quantitative zg profiling dataset. To quantify the detected metabolites acquired with zg sequence, standard curves of glucose (1.25 to 50 mM), fructose (3.65 to 10 mM), and glutamate (1.25 to 15 mM) were used. The glucose calibration was used for the quantification of all compounds, as a function of the number of protons of selected resonances except fructose and glutamate that were quantified using their own calibration curve. This allowed absolute quantification of 11 identified compounds, among them 9 were kept for statistical analysis to avoid redundancy with the other targeted analyses.

The assignments of metabolites were based on chemical shifts, signal multiplicities, intensity ratios, comparison with database values (the MeRy-B metabolomic database, http://bit.ly/meryb (Ferry-Dumazet et al. 2011), HMDB, www.hmdb.ca, BMRB, www.bmrb.wisc.edu and ChenomX NMR Suite 8.3 library (ChenomX Inc., Edmonton, Canada)) and with spectra of authentic compounds recorded in the same solvent conditions (in-house library), and by spiking the samples with the corresponding commercial compounds when doubt remained. 2D NMR experiments (COSY, COrrelation SpectroscopY; HSQC, Heteronuclear Single Quantum Correlation; HMBC, Heteronuclear Multiple Bond Correlation) with non-uniform sampling (NUS percent at 25 % and 128 points for COSY and HSQC and 512 points for HMBC) were acquired for selected samples for assignment verification.

***LC-QTOF-MS untargeted analysis of semi-polar metabolites***

LC-QTOF-MS profiling of aqueous methanol extracts containing 0.1% formic acid was performed from lyophilized powder (20 mg DW in 1 ml). An Ultimate 3000 HPLC (Dionex, Sunnyvale, CA, USA) was used to separate metabolites on a reversed phase C18 column (150×2.0 mm, 3 µm; Phenomenex, Torrance, CA, USA) using a 30 min linear gradient from 3% to 95% acetonitrile in water acidified with 0.1% formic acid. Metabolites were detected using a hybrid quadrupole/time-of-flight mass spectrometer (micrOTOF-*Q*, Bruker Daltonics, Bremen, Germany). Electrospray ionization in positive mode was used to ionize the compounds (500 V endplate offset, 4500 kV capillary voltage, 2.4 bar nebulizer gas, dry gas flow of 8 L/min at 190˚ C). Scan rate for ions at m/z range 50–1,500 was fixed at two spectra per second. Methyl vanillate was spiked in the extraction solvent and used as a control to check for injection. Five µL of all samples were thoroughly mixed together and used to produce a quality control sample (QC). A QC was injected after each set of 10 samples. The MS data were processed using XCMS (Smith et al. 2006). A total of 1519 features were detected. Data were filtered using: removal of features found in blank extraction, removal of features with coefficient of variation (CV) ratio calculated using QC samples above the 3^rd^ quartile, and removal of features characterized with *m/z* or retention time (RT) highly variable (RT_max_ - RT_min_ > 60 s, *m/z*_max_ - *m/z*_min_ > 0.025). Data were normalized using QCs and the sample weights. This resulted in 540. Metabolite identification was performed using the accurate mass UHPLC-LTQ-Orbitrap MS measurements (Thermo Fisher Scientific, Bremen, Germany) and targeted and dependent MS/MS data of a representative sample.

***Statistical Analyses***

All statistical analyses were performed using the R Software (http://www.r-project.org/), the R package mixOmics (Rohart et al. 2017) and the BioStatFlow online tool (biostatflow.org) which is based on R scripts. Two-way ANOVA with FDR correction was performed to highlight line status or water-treatment effects and interaction. The parameters used for partial least squares-discriminant analysis (PLS-DA) in BioStatFlow were adjusted to a 10-fold cross-validation (CV) to generate the model (and calculate the Q²) and 200-randomized permutations to estimate the robustness of the generated model. Some graphical outputs for PLS-DA were produced by mixOmics, using the same parameters than with BioStatFlow. An additional R script from Fu et al. (2017) was used to perform least absolute shrinkage and selection operator (LASSO) and sparse partial least square (sPLS) selection. Principal component analysis (PCA) and partial least square (PLS) were performed on data mean-centred and scaled to unit variance. All statistical analyses were performed on the data set in Online Resource 2 or subsets of this file. The data, whereas absolute (spectrophotometry and GC-FID) or relative (^1^H-NMR, HPLC and UPLC-fluo) contents were all normalized based on sample dry weights.

**References**

Allinne, C., Maury, P., Sarrafi, A., & Grieu, P. (2009). Genetic control of physiological traits associated to low temperature growth in sunflower under early sowing conditions. *Plant Science*, *177*(4), 349–359. doi:10.1016/j.plantsci.2009.07.002

Badouin, H., Gouzy, J., Grassa, C. J., Murat, F., Staton, S. E., Cottret, L., et al. (2017). The sunflower genome provides insights into oil metabolism, flowering and Asterid evolution. *Nature*, *546*(7656), 148–152. doi:10.1038/nature22380

Blanchet, N., Casadebaig, P., Debaeke, P., Duruflé, H., Gody, L., Gosseau, F., et al. (2018). Data describing the eco-physiological responses of twenty-four sunflower genotypes to water deficit. *Data in Brief*. Submitted

Bradford, M. M. (1976). A rapid and sensitive method for the quantitation of microgram quantities of protein utilizing the principle of protein-dye binding. *Analytical Biochemistry*, *72*, 248–254.

Deborde, C., Maucourt, M., Baldet, P., Bernillon, S., Biais, B., Talon, G., et al. (2009). Proton NMR quantitative profiling for quality assessment of greenhouse-grown tomato fruit. *Metabolomics*, *5*(2), 183–198. doi:10.1007/s11306-008-0134-2

Ferry-Dumazet, H., Gil, L., Deborde, C., Moing, A., Bernillon, S., Rolin, D., et al. (2011). MeRy-B: a web knowledgebase for the storage, visualization, analysis and annotation of plant NMR metabolomic profiles. *BMC plant biology*, *11*, 104. doi:10.1186/1471-2229-11-104

Fu, G.-H., Zhang, B.-Y., Kou, H.-D., & Yi, L.-Z. (2017). Stable biomarker screening and classification by subsampling-based sparse regularization coupled with support vector machines in metabolomics. *Chemometrics and Intelligent Laboratory Systems*, *160*, 22–31. doi:10.1016/j.chemolab.2016.11.006

Gibon, Y., Vigeolas, H., Tiessen, A., Geigenberger, P., & Stitt, M. (2002). Sensitive and high throughput metabolite assays for inorganic pyrophosphate, ADPGlc, nucleotide phosphates, and glycolytic intermediates based on a novel enzymic cycling system. *The Plant Journal*, *30*(2), 221–235. doi:10.1046/j.1365-313X.2001.01278.x

Gosseau, F., Blanchet, N., Varès, D., Burger, P., Campergue, D., Colombet, C., et al. (2018). Heliaphen, an outdoor high-throughput phenotyping platform designed to integrate genetics and crop modeling. *bioRxiv*, 362715. doi:10.1101/362715

Hendriks, J. H. M., Kolbe, A., Gibon, Y., Stitt, M., & Geigenberger, P. (2003). ADP-Glucose Pyrophosphorylase Is Activated by Posttranslational Redox-Modification in Response to Light and to Sugars in Leaves of Arabidopsis and Other Plant Species. *Plant Physiology*, *133*(2), 838–849. doi:10.1104/pp.103.024513

Jacob, D., Deborde, C., Lefebvre, M., Maucourt, M., & Moing, A. (2017). NMRProcFlow: a graphical and interactive tool dedicated to 1D spectra processing for NMR-based metabolomics. *Metabolomics*, *13*(4), 36. doi:10.1007/s11306-017-1178-y

Jelitto, T., Sonnewald, U., Willmitzer, L., Hajirezeai, M., & Stitt, M. (1992). Inorganic pyrophosphate content and metabolites in potato and tobacco plants expressing E. coli pyrophosphatase in their cytosol. *Planta*, *188*(2), 238–244. doi:10.1007/BF00216819

Nunes‐Nesi, A., Carrari, F., Gibon, Y., Sulpice, R., Lytovchenko, A., Fisahn, J., et al. (2007). Deficiency of mitochondrial fumarase activity in tomato plants impairs photosynthesis via an effect on stomatal function. *The Plant Journal*, *50*(6), 1093–1106. doi:10.1111/j.1365-313X.2007.03115.x

Patil, C., Calvayrac, C., Zhou, Y., Romdhane, S., Salvia, M.-V., Cooper, J.-F., et al. (2016). Environmental Metabolic Footprinting: A novel application to study the impact of a natural and a synthetic β-triketone herbicide in soil. *The Science of the Total Environment*, *566*–*567*, 552–558. doi:10.1016/j.scitotenv.2016.05.071

Poormohammad Kiani, S., Grieu, P., Maury, P., Hewezi, T., Gentzbittel, L., & Sarrafi, A. (2007). Genetic variability for physiological traits under drought conditions and differential expression of water stress-associated genes in sunflower (*Helianthus annuus* L.). *TAG. Theoretical and applied genetics. Theoretische und angewandte Genetik*, *114*(2), 193–207. doi:10.1007/s00122-006-0419-7

Rohart, F., Gautier, B., Singh, A., & Cao, K.-A. L. (2017). mixOmics: An R package for ‘omics feature selection and multiple data integration. *PLOS Computational Biology*, *13*(11), e1005752. doi:10.1371/journal.pcbi.1005752

Smith, C. A., Want, E. J., O’Maille, G., Abagyan, R., & Siuzdak, G. (2006). XCMS: processing mass spectrometry data for metabolite profiling using nonlinear peak alignment, matching, and identification. *Analytical Chemistry*, *78*(3), 779–787. doi:10.1021/ac051437y

Tompkins, D., & Toffaletti, J. (1982). Enzymic determination of citrate in serum and urine, with use of the Worthington “ultrafree” device. *Clinical Chemistry*, *28*(1), 192–195.
