# Supplementary material for: Metabolomic characterization of sunflower leaf allows discriminating genotype groups or stress levels with a minimal set of metabolic markers
Source: Metabolomics. 2019 Mar 30;15(4):56. doi: 10.1007/s11306-019-1515-4 (PMC6441456; doi:10.1007/s11306-019-1515-4)
Supplement: Supplementary file 3 — Supplementary material 3 (PPTX 239 kb) [file 11306_2019_1515_MOESM3_ESM.pptx]

## Slide 1
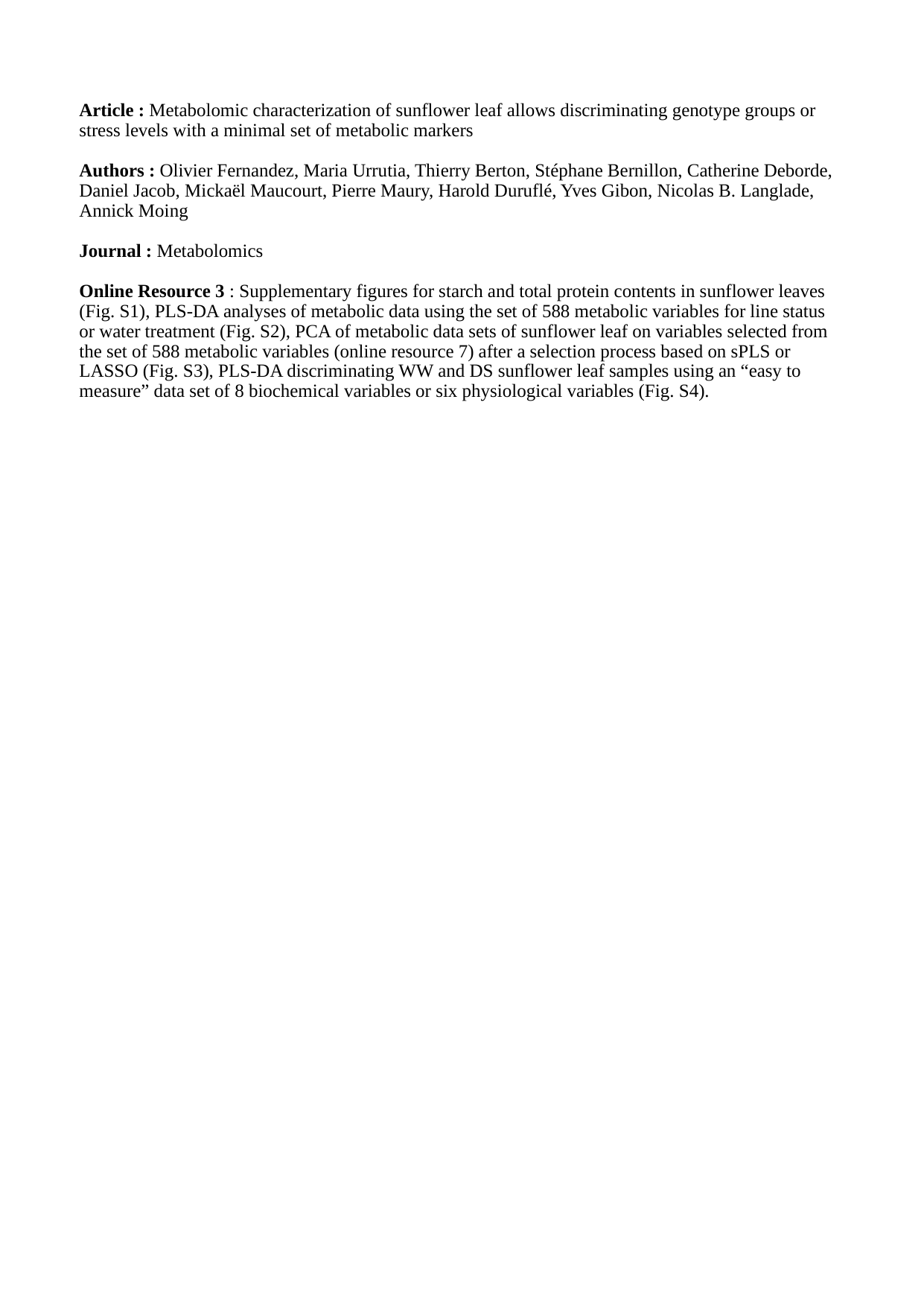

# Article : Metabolomic characterization of sunflower leaf allows discriminating genotype groups or stress levels with a minimal set of metabolic markers Authors : Olivier Fernandez, Maria Urrutia, Thierry Berton, Stéphane Bernillon, Catherine Deborde, Daniel Jacob, Mickaël Maucourt, Pierre Maury, Harold Duruflé, Yves Gibon, Nicolas B. Langlade, Annick Moing Journal : Metabolomics Online Resource 3 : Supplementary figures for starch and total protein contents in sunflower leaves (Fig. S1), PLS-DA analyses of metabolic data using the set of 588 metabolic variables for line status or water treatment (Fig. S2), PCA of metabolic data sets of sunflower leaf on variables selected from the set of 588 metabolic variables (online resource 7) after a selection process based on sPLS or LASSO (Fig. S3), PLS-DA discriminating WW and DS sunflower leaf samples using an “easy to measure” data set of 8 biochemical variables or six physiological variables (Fig. S4).

## Slide 2
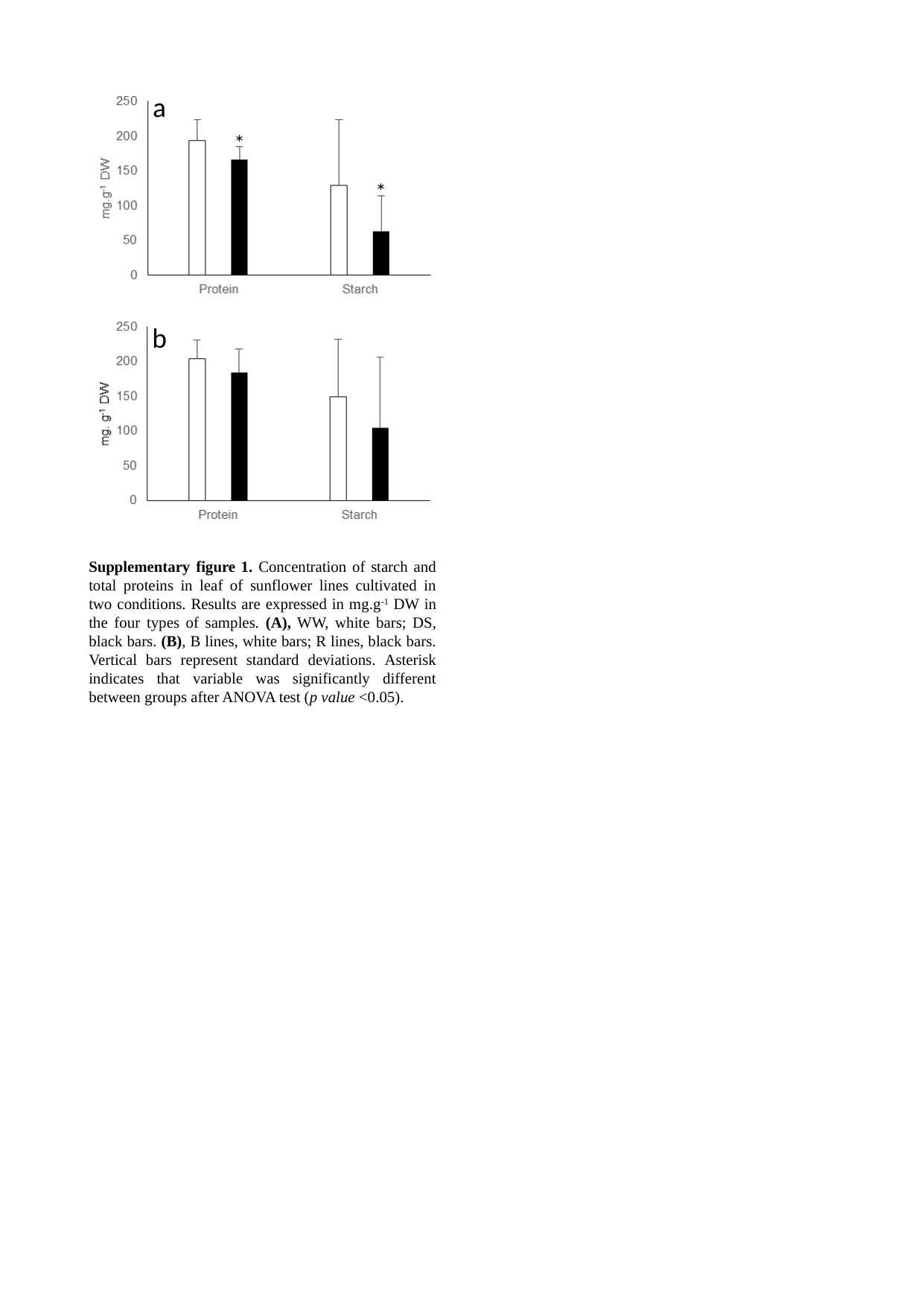

a
*
*
b
Supplementary figure 1. Concentration of starch and total proteins in leaf of sunflower lines cultivated in two conditions. Results are expressed in mg.g-1 DW in the four types of samples. (A), WW, white bars; DS, black bars. (B), B lines, white bars; R lines, black bars. Vertical bars represent standard deviations. Asterisk indicates that variable was significantly different between groups after ANOVA test (p value <0.05).

## Slide 3
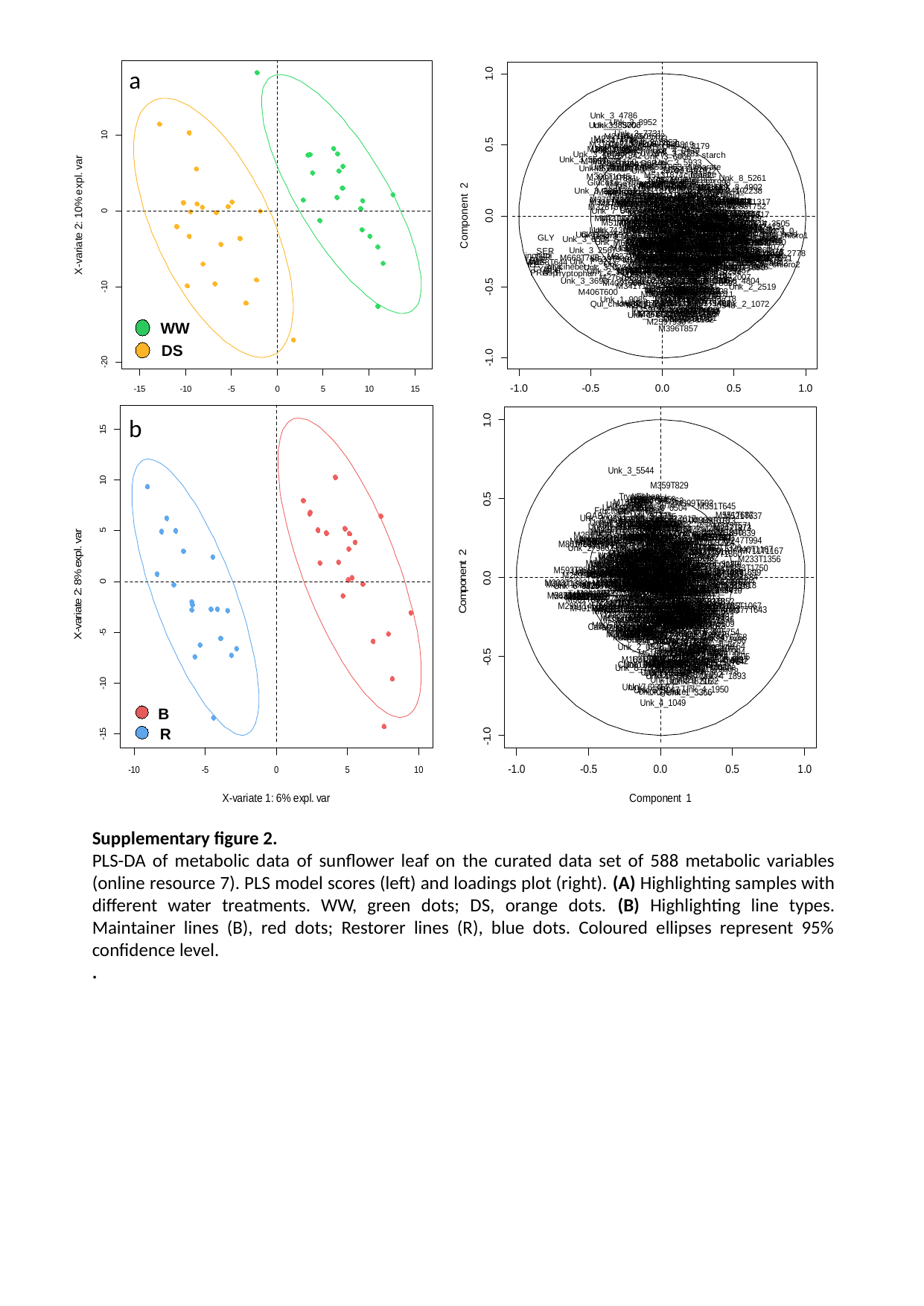

a
WW
DS
b
B
R
Supplementary figure 2.
PLS-DA of metabolic data of sunflower leaf on the curated data set of 588 metabolic variables (online resource 7). PLS model scores (left) and loadings plot (right). (A) Highlighting samples with different water treatments. WW, green dots; DS, orange dots. (B) Highlighting line types. Maintainer lines (B), red dots; Restorer lines (R), blue dots. Coloured ellipses represent 95% confidence level.
.

## Slide 4
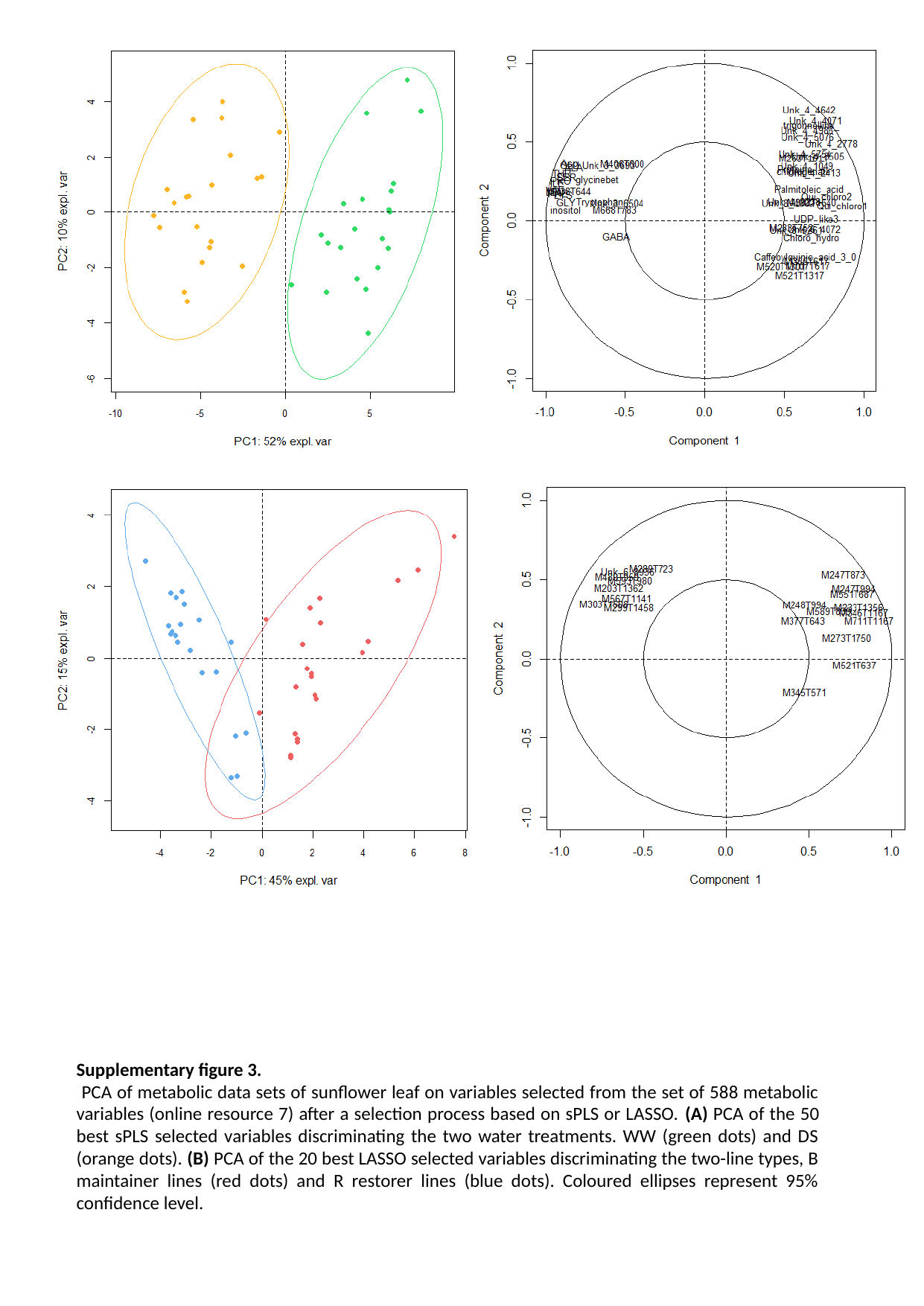

Supplementary figure 3.
 PCA of metabolic data sets of sunflower leaf on variables selected from the set of 588 metabolic variables (online resource 7) after a selection process based on sPLS or LASSO. (A) PCA of the 50 best sPLS selected variables discriminating the two water treatments. WW (green dots) and DS (orange dots). (B) PCA of the 20 best LASSO selected variables discriminating the two-line types, B maintainer lines (red dots) and R restorer lines (blue dots). Coloured ellipses represent 95% confidence level.

## Slide 5
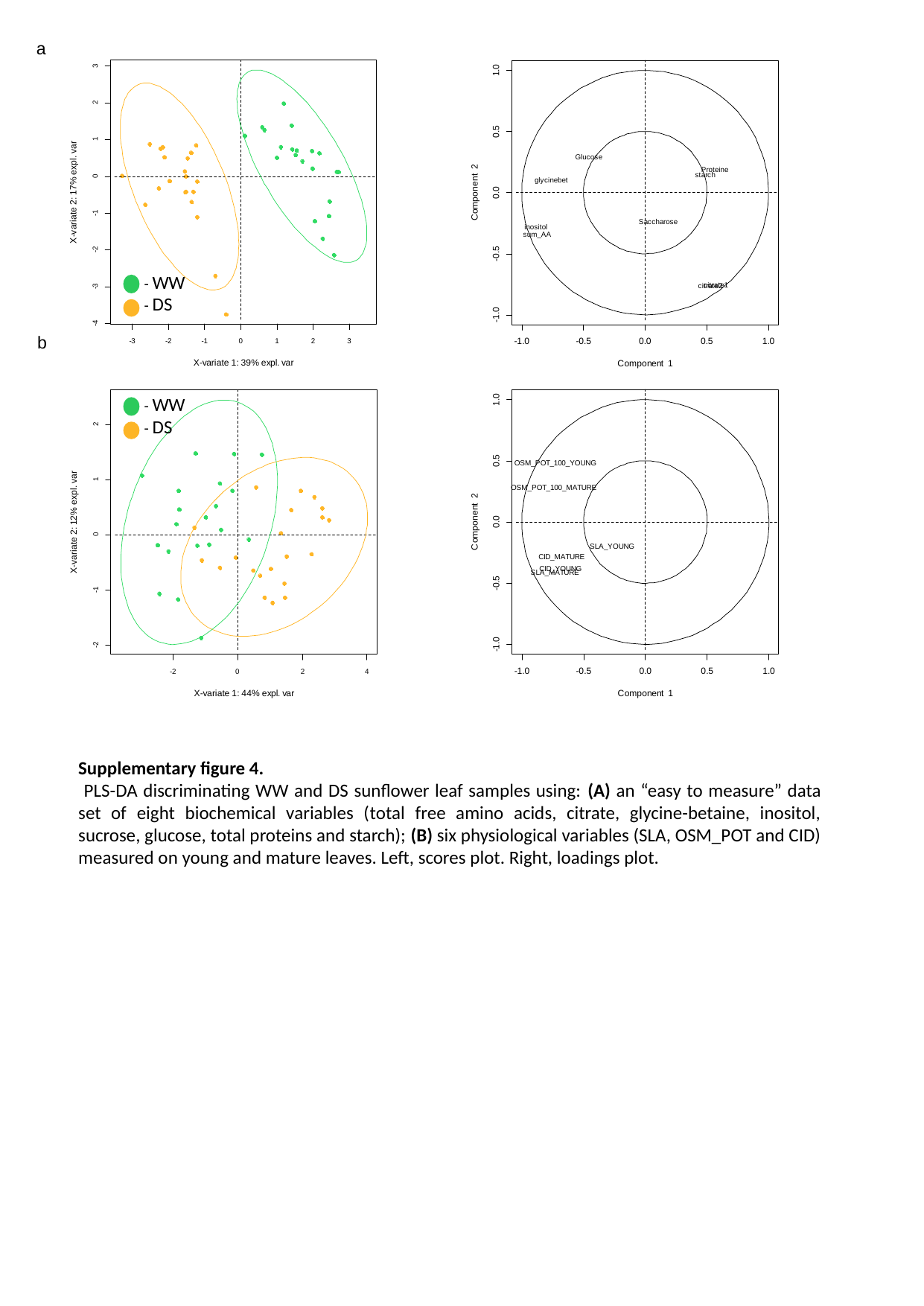

a
b
Supplementary figure 4.
 PLS-DA discriminating WW and DS sunflower leaf samples using: (A) an “easy to measure” data set of eight biochemical variables (total free amino acids, citrate, glycine-betaine, inositol, sucrose, glucose, total proteins and starch); (B) six physiological variables (SLA, OSM_POT and CID) measured on young and mature leaves. Left, scores plot. Right, loadings plot.
