# Supplementary material for: Metabolomic characterization of sunflower leaf allows discriminating genotype groups or stress levels with a minimal set of metabolic markers
Source: Metabolomics. 2019 Mar 30;15(4):56. doi: 10.1007/s11306-019-1515-4 (PMC6441456; doi:10.1007/s11306-019-1515-4)
Supplement: Supplementary file 5 — Supplementary material 5 (DOCX 30 kb) [file 11306_2019_1515_MOESM5_ESM.docx]

**Title**: Metabolomic characterization of sunflower leaf allows discriminating genotype groups or stress levels with a minimal set of metabolic markers

**Authors**: Olivier Fernandez, Maria Urrutia, Thierry Berton, Stéphane Bernillon, Catherine Deborde, Daniel Jacob, Mickaël Maucourt, Pierre Maury, Harold Duruflé, Yves Gibon, Nicolas B. Langlade, Annick Moing

**Journa**l: Metabolomics

**Online resource 5**: Annotation of methanolic extract metabolites by LC-MS and LC-MS-MS in positive mode

| **Metabolite Rt^a^**  **(min)** | **Metabolite [M+H]^+^**  ***m/z*** | **LC-MS adducts**  ***m/z*** | **LC-MS/MS fragments**  ***m/z*** | **Putative name** | **Class** | **Corresponding**  **molecular formula** | **Molecular formula calculated**  **[M+H]^+^**  ***m/z*** | **MSI level^b^** | **Putative Markers for** |
| --- | --- | --- | --- | --- | --- | --- | --- | --- | --- |
| 2.99 | 166.0860 | - | 120.0804 | Phenylalanine | Aminoacids | C_9_H_11_N_O2_ | 166.08626 | 2 | WT |
| 6.22 | 205.0968 | - | 188.0704 | Tryptophan | Aminoacids | C_11_H_12_N_2_O_2_ | 205.09715 | 2 | WT |
| 9.47 | 355.1018 | 377.0838 | 163.0389 | 5-O-Caffeoylquinic acid * | Cinnamic acids | C_16_H_18_O_9_ | 355.10236 | 1 | - |
| 9.83 | 341.0860 | - | 179.0334 | Aesculin | Coumarins | C_15_H_16_O_9_ | 341.08671 | 3 |  |
| 10.73 | 355.1013 | 377.0836 | 163.0387; 145.0280 | 3-O-Caffeoylquinic acid * | Cinnamic acids | C_16_H_18_O_9_ | 355.10236 | 1 | WT |
| 11.00 | 355.1020 | - | 163.0388 | 4-O-Caffeoylquinic acid * | Cinnamic acids | C_16_H_18_O_9_ | 355.10236 | 1 | - |
| 11.13 | 521.2012 | - | 485.1451 | Dihydroxy,trimethoxyflavone hexoside | Flavonoid | C_26_H_32_O_11_ | 521.20174 | 3 | LS |
| 13.02 | 465.1020 | 303.0493 | 303.0488 | Quercetin hexoside | Flavonoids | C_21_H_20_O_12_ | 465.10275 | 3 | - |
| 13.07 | 495.1124 | 517.0949 | 333.0593 | Pentahydroxy,methoxyflavone hexoside | Flavonoids | C_22_H_22_O_13_ | 495.11332 | 3 | - |
| 13.91 | 517.1322 | - | 499.1220 ; 471.3152 ; 325.0698;163.0387 | 3,5-Dicaffeoylquinic acid * | Cinnamic acids | C_25_H_24_O_12_ | 517.13405 | 1 | - |
| 14.33 | 517.1333 | - | 499.1223 | 3,4-Dicaffeoylquinic acid * | Cinnamic acids | C_25_H_24_O_12_ | 517.13405 | 1 | - |
| 14.94 | 251.1638 | - | 233.1531; 165.0863 ; 153.0864; 127.1113 | Heliannuol A or D | Sesquiterpenoids | C_15_H_22_O_3_ | 251.16417 | 3 | WT |
| 15.89 | 377.1592 | 399.1413 | 359.1478; 277.1064 ; 259.0958 ; 241.0854 ; 231.1008; 213.0904 | Anhydridoniveusin | Sesquiterpenoids | C20H24O7 | 377.15948 | 3 | - |
| 16.98 | 379.1743 | 401.1573 | 361.1638 ; 279.1220 ; 261.1115 ; 243.1009 ; 231.1010; 225.0905; 215.1061; 197.0957 | Niveusin C or Hydroxyleptocarpin | Sesquiterpenoids | C_20_H_26_O_7_ | 379.17513 | 3 | - |
| 17.04 | 331.0803 | - | 316.0569 ; 301.0336; 298.0466 | Trihydroxy,dimethoxyflavone | Flavonoids | C_17_H_14_O_7_ | 331.08123 | 3 | - |
| 17.24 | 409.1846 | 431.1675 | 291.1220; 273.1115; 259.0958; 241.0854; 213.0905 | 3-O-Methylniveusin A | Sesquiterpenoids | C_21_H_28_O_8_ | 409.18569 | 3 | - |
| 17.24 | 361.0909 | - | 346.0672; 331.0439; 328.0571; 261.1116 | Trihydroxy, trimethoxyflavone I | Flavonoids | C_18_H_16_O_8_ | 361.09179 | 3 | - |
| 17.97 | 361.0909 | - | 346.0674; 331.0440; 328.0570; 313.0337 | Trihydroxy,trimethoxyflavone II | Flavonoids | C_18_H_16_O_8_ | 361.09179 | 3 | - |
| 19.39 | 345.0958 | - | 330.0723 ; 329.0647; 315.0490 ; 312.0621 ; 297.0386 ; 284.0673 | Dihydroxy,trimethoxyflavone I | Flavonoids | C_18_H_16_O_7_ | 345.09688 | 3 | - |
| 19.72 | 345.0958 | - | 330.0723; 315.0492 ; 312.0623 | Dihydroxy,trimethoxyflavone II | Flavonoids | C_18_H_16_O_7_ | 345.09688 | 3 | LS |
| 22.19 | 353.2678 | - | 335.2571; 317.2468; 279.2313; 261.2205; 243.2101 | Linolenoyl-glycerol I | Lipids | C_21_H_36_O_4_ | 353.26864 | 3 | - |
| 22.48 | 353.2679 | - | 335.2574; 317.2469; 279.2313; 261.2206; 243.2102 | Linolenoyl-glycerol II | Lipids | C_21_H_36_O_4_ | 353.26864 | 3 | - |
| 27.15 | 301.2154 | - | 283.2051; 255.2103; 227.1791 | Retinoic acid | Lipids | C_20_H_28_O_2_ | 301.21621 | 3 | - |

^a^: Rt were measured with the UHPLC-LTQ-Orbitrap system

^b^: According to Sumner et al. 2007 (Metabolomics 3:211)

* : compared with an authentic standard

LS: line status; WT: water treatment
